# Bioequivalence: tried and tested

**Published:** 2010-04

**Authors:** Robert Schall, Laszlo Endrenyi

**Affiliations:** Department of Mathematical Statistics and Actuarial Science, University of the Free State, Bloemfontein, South Africa; Department of Pharmacology, University of Toronto, Ontario, Canada

Two drug products are considered bioequivalent ‘if their bio-availabilities ... are similar to such a degree that their effects, with respect to both efficacy and safety, will essentially be the same’.^1^ The bioequivalence of two drug products is generally demonstrated through a clinical study in healthy volunteers, the so-called bioequivalence study. If bioequivalence is shown for two drug products, therapeutic equivalence of the drug products is implied. Chow and Liu^2^ call this assumption, namely that bioequivalence implies therapeutic equivalence, the ‘fundamental bioequivalence assumption’.

Most drug products on the market today have been subjected to bioequivalence assessment at various stages in their development. As is well known, generic drug products require the demonstration of bioequivalence to the relevant innovator product for regulatory approval. What is perhaps less well known is that most innovator products, too, require some form of bioequivalence testing. New drugs typically undergo pharmacokinetic dose-proportionality studies, and drug–drug and drug–food interaction studies, all of which use the bioequivalence concept. The site of development and production of the drug product could be changed. Most importantly, when the innovator formulation to be marketed is different from the formulation used previously in pivotal efficacy trials, as is often the case, bioequivalence of the marketed formulation to the clinical trial formulation must be shown. In this sense, many innovator drug products on the market are in fact ‘generic copies’ of the clinical trial formulation for which therapeutic efficacy and safety had been shown in patients.

Consumers of drug products, therefore, of both generic and innovator products, need assurance on the question whether bioequivalence implies therapeutic equivalence. All drug manufacturers of either generic or innovator products need to know whether the bioequivalence concept and bioequivalence methodology serve their products well during development. On both these questions relatively recent developments have shed some light.

## History of the bioequivalence concept

Public concern and ongoing discussion about bioequivalence started in the early 1970s with reports about digoxin intoxications. At the time, generic digoxin formulations were increasingly prescribed in the United States, and a change in the manufacturing process of a company in Great Britain led to an unintentional increase in the bioavailability of one brand of digoxin tables.^3^,^4^ It became clear that drug products that are pharmaceutically equivalent, that is, products that contain the same drug in the same dose, are not necessarily bioequivalent.^5^

Over the years, various regulatory guidelines on the design, conduct and statistical analysis of bioequivalence studies have been published. Many years of research, discussion and controversy culminated in the seminal Food and Drug Administration’s (FDA) 1992 guidance^6^ on the statistical analysis of bioequivalence studies. That guidance established such well-known concepts as the pharmacokinetic characteristics for rate and extent of drug absorption and the statistical decision rule for the demonstration of bioequivalence (90% confidence interval for the test/reference ratio of mean bioavailability must fall completely in the bioequivalence acceptance range of 80–125%.)

## Switchability of drug products: the individual bioequivalence intermezzo

With the publication of the 1992 FDA guidance,^6^ one might have thought that agreement had been reached, finally, among researchers and regulators on the central concepts of bioequivalence. Ironically, almost exactly around that time, the new concept of individual bioequivalence^7^ was formulated and sparked a new era of research and discussion, and probably more controversy than ever before.

Two US biostatisticians, Anderson and Hauck,^7^ pointed out that the traditional way of bioequivalence assessment, as circumscribed, for example, in the contemporary 1992 FDA guidance, ensured merely that the bioavailability of two drug products was similar (‘equivalent’) on average. They raised the following clinically very relevant question: does equivalence of average bio-availability, which they termed average bioequivalence, ensure that the bioavailability of two drug products is equivalent in individual patients? In other words, does average bioequivalence imply switchability of drug products in individual patients?

Following the groundbreaking article of Anderson and Hauck, numerous statistical approaches to individual bioequivalence were published (Schall^8^ provides a unified view of most of the approaches; see also the reviews of Hauschke, Steinijans and Pigeot^9^ and Chow and Liu^2^). Eventually, in 2001, the individual bioequivalence concept was adopted in an FDA guidance.^10^ However, ‘responses [to the guidance] were doubt-filled as to whether the new bioequivalence criteria really provided added value compared to average bioequivalence’.^9^

Crucially and rather illuminating on the question of the general validity of the fundamental bioequivalence assumption was the observation that ‘there has been no published evidence of clinical failure with a formulation demonstrated to be equivalent to the reference product under average bioequivalence’.^11^,^12^ Individual bioequivalence was called a ‘theoretical’ solution to a ‘theoretical’ problem.^13^ In response to the widespread criticism and doubts, in 2003 the FDA omitted the individual bioequivalence concept from a subsequent guidance,^14^ and returned, almost full circle, to the conventional (average) bioequivalence concept of the 1992 guidance.^6^

## Bioequivalence and therapeutic equivalence

The concept of individual bioequivalence proved to be an intermezzo but it proved to be useful after all. Critical and clinically relevant questions were asked about the traditional concept of average bioequivalence, and this concept emerged strongly. In particular, the crucial question, namely whether bioequivalence implied therapeutic equivalence, was answered in the affirmative. In a highly competitive market and litigious society such as the United States one would expect cases of therapeutic inequivalence of bioequivalent products to be publicised quickly and widely. Nevertheless, Rheinstein^15^ could state in 1990 that ‘To date, there is no evidence of therapeutic inequivalence in a properly manufactured generic drug which has been approved as bioequivalent by the FDA’.

A literature search conducted by Gould^11^ 10 years later to answer the question whether average bioequivalence implied switchability of drug products in practice came to the same conclusion: essentially no evidence of therapeutic failure of bioequivalent products could be found. Chow and Liu^2^ reported how in the United States innovator companies file citizen petitions in order to convince the regulatory agency (FDA) that a generic copy of a brand-name drug will not achieve therapeutic equivalence even if the generic has been shown to be bioequivalent to the brand name drug. Those authors cite no case of a generic that has been approved as bioequivalent by the FDA but has been shown to be therapeutically inequivalent to the innovator. [However, it should be noted that there are classes of drugs whose safety are particularly sensitive to the conditions of administration. For example, responses to immunosuppressants, and the contrasts between different drug products can vary with time after transplantation, the target organ, ethnicity and concomitant disease conditions (e.g. diabetes) of the patients.^16^]

In summary, the fundamental bioequivalence assumption, namely that bioequivalent products are therapeutically equivalent and can be used interchangeably, has survived strong scrutiny; scrutiny that was *inter alia* a by-product of the discussion of and research on the individual bioequivalence concept. The conventional concept of average bioequivalence seems to have served the consumers of drugs rather well.

## Highly variable drugs and widening the bioequivalence acceptance range, or scaling

What about the concerns that the producers of drugs might have with the bioequivalence concept? It is well known that the conventional approach of average bioequivalence, with an 80–125% acceptance range, can make it very difficult to show bioequivalence for drug products with highly variable bioavailability (so-called ‘highly variable drugs’ or drug products). For such drugs, sample sizes of 100 subjects and higher can be required to demonstrate bioequivalence. ‘A feature of the difficulties involving the determination of bioequivalence of highly variable drugs is that, under typical conditions, a drug product may not be found bioequivalent to itself.’^17^ This is clearly unsatisfactory, in particular to producers of drug products who face inordinate costs when conducting bioequivalence studies for highly variable drugs.

A potential solution to the problem of highly variable drugs is suggested by the observation that most highly variable drugs have a wide therapeutic index. If such a drug indeed has a wide therapeutic index, it should be clinically acceptable to widen the bioequivalence acceptance range for it. Various ways of appropriately widening the acceptance range for highly variable/wide therapeutic-index drugs have recently been discussed and investigated.^17^,^18^ The approach that currently seems to be favoured by FDA scientists and researchers in the field is that of scaled average bioequivalence.^17^,^18^

Without going into the methodological and statistical details, the scaled average bioequivalence concept effectively widens the conventional acceptance range for bioequivalence, namely 80–125%, proportionally to the within-subject standard deviation of the bioavailability of the reference product. Therefore, the more variable the bioavailability of the reference drug product, the wider the effective acceptance range for bioequivalence. Interestingly, the basic concept of scaling the bioequivalence criterion had already been proposed early in the development of characteristics for individual bioequivalence,^8^,^19^,^20^ so that the scaled average bioequivalence concept can be viewed as another by-product of the research into individual bioequivalence. While there still are some problems with the scaled average bioequivalence concept,^17^ at present it seems to be the most promising and practical approach for handling the problem of highly variable drugs in bioequivalence.

## Narrow therapeutic index drugs and narrowing the bioequivalence acceptance range

The mirror image of highly variable drugs with wide therapeutic index is narrow therapeutic-index drugs whose variability typically is low. If it is reasonable to widen the bioequivalence acceptance range for highly variable drugs with wide therapeutic index, it seems equally reasonable to narrow the bioequivalence acceptance range for drugs with low variability and narrow therapeutic index. Such a narrowing of the bioequivalence acceptance range for narrow therapeutic-index drugs could increase assurance, particularly of the safety of generics in this drug class, without imposing an undue burden, financial or otherwise, on the sponsors of bioequivalence studies for such drugs. Indeed, the new European bioequivalence guideline^21^ envisages that ‘in specific cases of products with narrow therapeutic index, the acceptance interval for AUC should be tightened to 90–111.11%’.

## Conclusion

The standard approach to bioequivalence assessment, namely conventional average bioequivalence, has proven itself under strict scrutiny over more than 20 years. Drug products that under a proper regulatory regime have been approved as bioequivalent to a reference product can generally be assumed to be therapeutically equivalent to that reference product. The bioequivalence limits could be widened relative to the conventional acceptance range of 80–125% for handling the problem of highly variable drugs, and could be narrowed for narrow therapeutic-index drugs. For highly variable drugs, scaled average bioequivalence provides an alternative, effective approach to the comparison of drug products.
